# “Miss, I want itchy medicine”: Understanding what, why, and how antibiotics are used in Central Java province, Indonesia through the drug bag method

**DOI:** 10.1371/journal.pgph.0005933

**Published:** 2026-03-05

**Authors:** Soe Yu Naing, Mira L. Schneiders, Dhini Ningrum, Tri Nurdianti, Juliëtte Severin, Koen Peeters Grietens, Jaap A. Wagenaar, Anis Karuniawati

**Affiliations:** 1 Division of Infectious Diseases and Immunology, Department of Biomolecular Health Sciences, Faculty of Veterinary Medicine, Utrecht University, Utrecht, The Netherlands; 2 Department of Public Health, Institute of Tropical Medicine, Antwerp, Belgium; 3 Department of Medical Microbiology and Infectious Diseases, Erasmus MC University Medical Center Rotterdam, Rotterdam, The Netherlands; 4 Department of Microbiology, Faculty of Medicine, Universitas Indonesia, Jakarta, Indonesia; Burnet Institute, AUSTRALIA

## Abstract

Antibiotic use is shaped by access, knowledge, norms, and practices, yet remains poorly understood in many community settings worldwide. To address these gaps, we investigated community antibiotic use in six villages across Sukoharjo, Karanganyar, and Klaten districts of Central Java province in Indonesia. This study focused on three key questions: (i) which antibiotics are accessible in community settings; (ii) how do community members recognize and understand these antibiotics; and (iii) what social, economic, cultural, and contextual factors drive antibiotic use. Between April and May 2024, 36 drug bag interviews, in which locally available antibiotics were purchased, compiled, and used in household pile-sorting activities, were conducted with community members. Complementary ethnographic fieldwork included simulated patient visits, informal conversations, and participant observations in community settings and at local pharmacies and shops (*Warungs*). This study found that knowledge of antibiotics and Indonesian medicine labels was generally low, while access to antibiotics without prescription, particularly from pharmacies and *Warungs*, was high. *Access* antibiotics, such as amoxicillin and tetracycline, were the most commonly used and recognized, while *Watch* antibiotics, including aminoglycosides, were also popular for self-medication, especially in topical forms. Common reasons for self-medication included convenience, ease of access, and lower cost. Practices such as stopping treatment early, repurposing antibiotics, and sharing them among family members were commonly adopted. This study identified multiple intersecting factors influencing antibiotic use in community settings. The findings highlight that effective antibiotic stewardship requires not only policies to restrict non-prescription sales but also interventions that address the social, economic, and cultural drivers of self-medication.

## Introduction

Antibiotics are essential medicines that save lives. However, while access to antibiotics is crucial for treating infections, their widespread use has jeopardized their long-term effectiveness. In fact, an estimated 80% of antibiotics are used in community settings globally, and nearly half of the global population obtains them without a prescription [1,2]. Especially in low- and middle-income countries (LMICs), where over-the-counter (OTC) antibiotics are more affordable and accessible than formal healthcare services, they are frequently used as quick remedies for everyday ailments [3]. A systematic review and meta‐analysis of OTC antibiotic dispensing in community pharmacies found that, overall, 62% of human antibiotics were sold without prescription globally [4]. In LMICs, the availability of antibiotics without a prescription remains particularly high despite existing regulations that are weakly enforced. For example, Indonesia, Syria, and Saudi Arabia have the highest rates of non-prescription antibiotic supply (91%, 87% and 85%, respectively), significantly above the global pooled estimate of 62% and the Asian regional estimate of 65% [4]. This high availability often coexists with settings where access is limited due to rurality and frequent stock-outs, resulting in precarious access [5,6]. Together, these conditions drive informal antibiotic access in LMICs and this frequent use reflects high levels of antimicrobial resistance (AMR) in these countries. Critically, these AMR rates, along with high usage of antibiotics, are rooted in structural factors such as poor sanitation, overcrowding, and weak public health infrastructure that create living environments where infections spread readily.

To safeguard antibiotic efficacy, the WHO global action plan on AMR calls for optimizing antimicrobial use across human, animal, and agricultural sectors, alongside the establishment of robust surveillance systems [7]. However, current surveillance mechanisms, such as the Global Antimicrobial Resistance and Use Surveillance System (GLASS), primarily capture data from hospitals, insurance claims, sales figures, or formal community pharmacy records. As a result, comprehensive antibiotic use data on community level remains generally lacking. In addition, these monitoring systems rarely include antibiotic consumption at local level through informal healthcare markets, which dominate antibiotic distribution in many LMICs. Consequently, there is a significant gap in understanding real-world patterns of antibiotic access and use, particularly at the household level.

To bridge this evidence gap, Dixon et al. developed the participatory ‘Drug Bag’ method, which uses physical antibiotic samples to document household and farm antibiotic use, recognition and accessibility [8]. Pilot testing of this ethnographic tool in Myanmar, Malawi, Uganda, and Zimbabwe demonstrated its suitability in resource‐limited settings, where monitoring of antibiotic use is not adequately established [8–10]. Following these pilot studies, the ‘Drug Bag’ method was adopted in research studies conducted in rural Malawi [11], Burkina Faso [12], and Tanzania [13]. These findings confirm the method’s ability to collect accurate usage volumes, local recognizability, frequency of use, and diversity of antibiotic types available in a given community. Moreover, ‘Drug Bag’ interviews can help to identify community health concerns, health‐seeking behaviours, and the informal supply‐chain dynamics that shape antibiotic access. Thus, the ‘Drug Bag’ approach is a suitable research tool for gaining a nuanced understanding of community-level antibiotic use, as it captures not only which antibiotics are available, but also social and cultural factors that influence their use.

Understanding community-level antibiotic practices is especially crucial in countries like Indonesia, the most populous country in Southeast Asia (270 million), with a high and growing demand for veterinary and human use of antibiotics. Additionally, although regulations exist, enforcement of antibiotic stewardship and regulations remain limited, highlighting the importance of understanding local practices in order to develop tailored antimicrobial stewardship interventions [14].

To address these knowledge gaps, the COINCIDE study (‘Impact of reducing colistin use on colistin resistance in humans and poultry in Indonesia’) was developed to investigate community‐level use of colistin, a last‐resort antibiotic, and other medically important antibiotics in community settings and on farms in Central Java province, Indonesia [15]. Central Java province was deliberately chosen for the study due to the availability of pre-ban poultry and community data from that region in 2014. Indonesia’s recent ban on colistin in agriculture provides a unique intervention to examine its impact on colistin resistance and to study changes in antibiotic access and use [15,16]. Understanding these dynamics is important because antibiotic use in the community remains widespread and poorly documented.

A systematic review on antibiotic use in humans in Indonesia by Limato et al. (2022) also reported that evidence on antibiotic practices in Indonesia is fragmented with significant gaps in private and informal healthcare sectors [17]. Further, a mixed-method study in West Java and South Kalimantan found that antibiotics were dispensed without a prescription in 69% of simulated-patient visits, driven by strong patient demand, unqualified sellers, business interests, and weak regulatory enforcement [18,19]. These findings highlight the need for a deeper understanding of antibiotic access, usage patterns, public knowledge, and the behavioural drivers of community-level antibiotic use.

Addressing these challenges and evidence gaps, this study focused on three research questions: (i) which antibiotics are accessible in community settings?; (ii) how do community members recognize and understand these antibiotics? and (iii) what social, economic, cultural and contextual factors drive antibiotic use in these settings?

## Methods

### Study context and setting

This ethnographic research was conducted between 8 April and 24 May 2024 as part of the COINCIDE project [15] in Central Java province, Indonesia, focusing on six villages across the districts of Sukoharjo, Karanganyar, and Klaten. The COINCIDE study ran for three years, with about 14 months of fieldwork. The drug‑bag activity was the final component, supported by earlier ethnographic work including participant observation, informal conversations in communities and farms, and interviews with primary healthcare staff. The study locations were previously studied in 2014 for antibiotic use and resistance in humans and poultry. These districts were selected to maintain continuity with earlier findings and because they remained active sites for COINCIDE fieldwork, including human and poultry sampling, and ethnographic work at primary healthcare centers. Village selection followed three criteria: (1) inclusion within designated sub-districts under participating primary healthcare facilities (Puskesmas), (2) presence of an active village midwife, and (3) at least one household engaged in small-scale chicken farming. Local stakeholders, including Puskesmas heads, midwife coordinators, nurses, and village midwives, were engaged in a participatory process to identify suitable villages, drawing on seven months of collaboration with the research team. Stakeholders were briefed on the drug bag study’s objectives and methods, and their recommendations informed final village selection, which was confirmed during consultative meetings in each sub-district. Table 1 provides an overview of the six selected villages and the dates of drug bag interviews conducted in each district.

**Table 1:** Study sites and dates of drug bag interviews per district.

No	Location			Dates of drug bag interviews
	District	Subdistrict	Village	
1	Sukoharjo	Bendosari	Mertan	15 – 27 April 2024
			Sugihan	
2	Karanganyar	Mojogedang	Sewurejo	29 April – 10 May 2024
			Pojok	
3	Klaten	Karanganom	Gempol	13 – 24 May 2024
			Ngabeyan	

In terms of study context, the study region is predominantly ethnic Javanese (97–98%) and Islam is the dominant religion (97.4%), with Christian, Buddhist, and Hindu minorities [20]. Javanese is the primary spoken language, while Bahasa Indonesia is used for formal communication [21]. Economically, residents engage in a combination of formal employment such as civil service, factory work, and private sector jobs, and informal livelihoods, including agriculture, livestock farming, and small-scale trade [22]. Households commonly cultivate rice, farm vegetables, and raise livestock such as cattle, goats, chickens, and buffalo. Community water access is variable across districts, including municipal water, wells or rainwater harvesting (unpublished data, field notes from COINCIDE study). Food practices are shaped by Islamic dietary norms and local customs. Meals are typically halal and shared communally, with local reports of food-borne infections and outbreaks associated with community events (unpublished data, field notes from COINCIDE study). Health services are accessed through either village-level midwives or Puskesmas, but specialized care requires travel to sub-district or district centers. In addition to formal health facilities, *Warungs* and local pharmacies are also commonly accessed by the general population as first-line points for health services and medicine acquisition at the community level in Indonesia. Non-communicable diseases like hypertension and diabetes are increasingly reported, and many residents use a mix of biomedical and traditional remedies (unpublished data, field notes from COINCIDE study). Among livestock, routine use of antibiotics for prevention and treating sick animals is common, while traditional herbs are also sometimes used to improve animal health in small-scale chicken farms (unpublished data, field notes from COINCIDE study). These social, cultural, and economic contexts provide a compelling rationale for selecting these locations as the research sites to examine community-level practices regarding antibiotic use, in addition to the aforementioned COINCIDE study criteria [15].

### Data collection

#### Drug bag method.

This study employed the drug bag method interviews, a qualitative approach adapted from Dixon et al (2016) [8], which combines anthropological pile-sorting techniques with in-depth interviews (IDIs). This method begins with researchers identifying and purchasing all unique brands of antibiotics available from formal and informal sources in the study area (i.e., pharmacies and village shops within the selected villages). The first batch of antibiotics used for the initial drug bag visit was obtained using a collaborating general practitioner’s prescription for research purposes; subsequently, additional antibiotics were purchased through simulated patient visits, also known as mystery patients, without presenting a prescription, following participant interviews, with these findings reported in Table 3. These antibiotics are then labelled and compiled into a single “drug bag”, which is brought to participants’ homes for the interviews.

During the interview, participants are presented with the contents of the drug bag and asked to sort the antibiotics into different ‘piles’ in response to a series of structured questions. Each question prompts the creation of a unique pile, which is documented through photographs and detailed fieldnotes. Further methodological details are provided in Dixon et al. [8].

**Fig 1:**
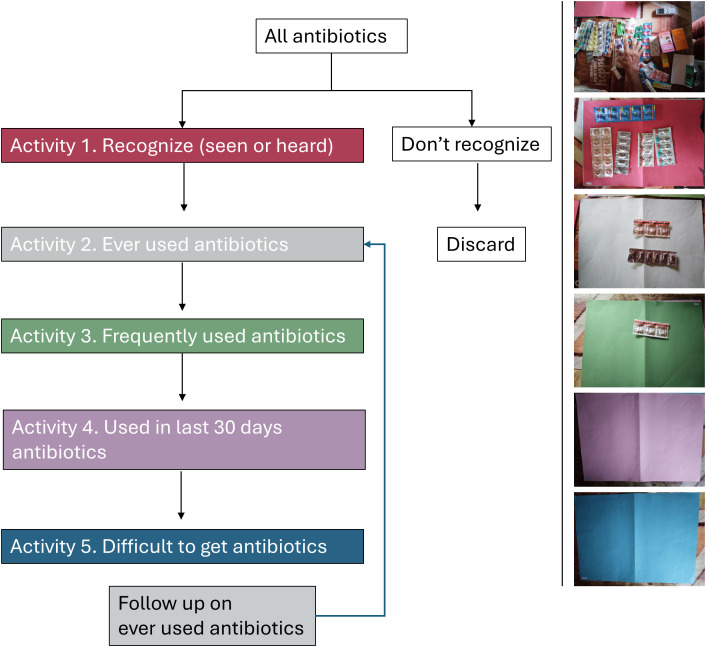
Summary of pile-sorting activity. Diagram showing the progression of pile-sorting activity questions for each drug bag interview (left) and examples of research outputs for each drug bag activity (right).

**Fig 2:**
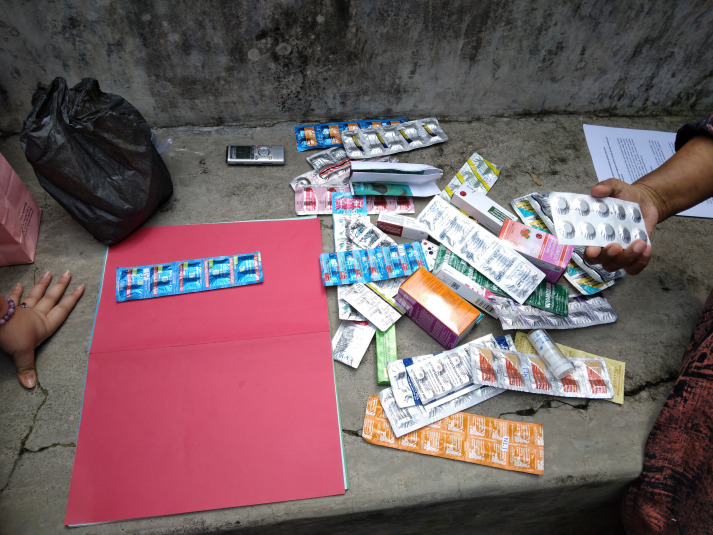
Picture of the drug bag interview in action. The drug bag method data collection in action in Ngabeyan village, Klaten district, Central Java province, Indonesia.

A topic guide, adapted from Dixon et al. (2016) [8], was developed, pilot tested, and used across all drug bag interviews (see S1 File). The topic guide was structured into three main sections. The first section collected basic demographic information, including age, sex, occupation, education level, household composition, and the participant’s role within the household. The second section focused on the medicine sorting activity and included five structured questions designed to guide participants through the pile-sorting process (see Table 2). The final section comprised broader questions related to antibiotic use in animals, general knowledge of antibiotic medicines, and familiarity with medicine labels commonly used in Indonesia. A visual overview of the drug bag interview flow is provided in Fig 1, and an example photo of data collection is shown in Fig 2.

**Table 2:** Five questions pertaining to the medicine sorting activities conducted during drug bag interviews.

Medicine sorting activity	Guiding interview question
1 (among all medicines in the drug bag)	**Which of these medicines have you seen or heard of before?** Please place them together in a pile.
2 (among ‘recognise’ pile)	**Which of these medicines have you or one of your household members ever used before in your household?** Please place them together in a pile.
3 (among ‘ever used’ pile)	**Which of these medicines do you use frequently when someone in your household is sick?** Please place them together in a pile.
4 (among ‘ever used’ pile)	**Which of these medicines did you or one of your household members use in the last 30 days (i.e., 1 month)?** Please place them together in a pile.
5 (among ‘ever used’ pile)	**For each of these medicines, has there ever been a time when you have needed this medicine but could not get it?** Please place them together in a pile.

Each household was visited once, with no repeat interviews being conducted, and transcripts were not returned to participants for comment. Drug bag interviews were audio-recorded, and sorted ‘piles’ were photographed.

#### Ethnography.

In addition to the drug bag interviews, ethnographic fieldwork was carried out throughout the data collection period. Ethnographic fieldwork included simulated patient visits, informal conversations, and participant observations in community settings and at local pharmacies and shops (*Warungs).* Ethnographic fieldwork involved informal conversations that emerged spontaneously during daily interactions with community members, offering perspectives into everyday health practices and how antibiotics are understood and used. Participant observations were conducted in local pharmacies and community shops (*Warungs*), allowing the researcher to observe purchasing practices, advice-giving, and interactions between sellers and community members in their natural contexts.

Researchers spent between one to two weeks in each village, including 2–3 days of coordination and planning and 3–5 days of ethnographic fieldwork and data collection. All components of data collection were conducted by the same two local researchers (DN and TN; see ‘Research Team and Reflexivity’ for details).

#### Simulated patient visits.

**Fig 3:**
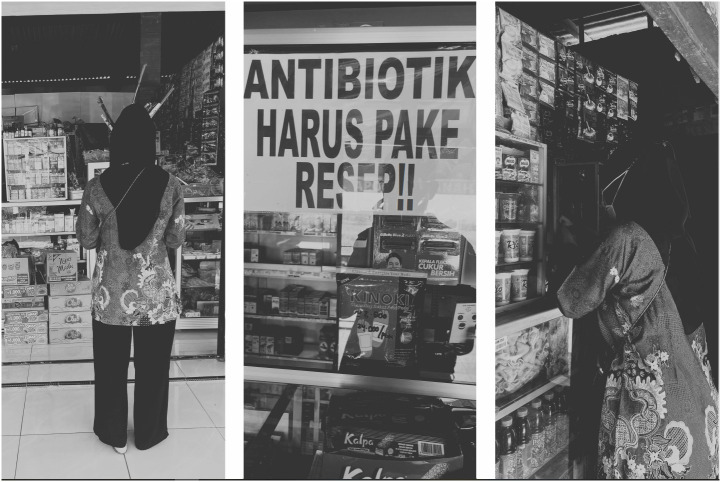
Picture of the local pharmacy window. Simulated patient visits to local pharmacies and *Warungs* (“Antibiotik harus pake resep” written in Bahasa Indonesia in the window at a local pharmacy means “Antibiotics require a prescription”).

Simulated patient visits, also sometimes known as mystery client visits, involved the local research team (DN and TN) posing as real patients/clients to study antibiotic providers’ behaviour in a naturalistic setting (local pharmacies and *Warungs*) without revealing their researcher identity (Fig 3). This method allowed the researchers to observe actual dispensing practices and communication patterns, while minimizing the risk of social desirability or observer bias.

To conduct simulated patient visits and to obtain physical samples of all antibiotics available in each village, pharmacies and *Warungs* were selected using a combination of sources: official pharmacy lists provided by Puskesmas staff, searches conducted via Google Maps, and information gathered through informal conversations with community members. Researchers then posed as fake patients during their pharmacy or *Warung* visit, describing symptoms such as sore-throat, urinary tract infection, itchiness with watery blisters (dermatitis), which were identified as common reasons for antibiotic prescribing during preliminary field visits conducted prior to data collection. When asked, researchers claimed that these symptoms had been present for more than three days.

During simulated patient visits, informal conversations (ICs) and participant observations (POs) with both antibiotic consumers (i.e., community members) and dispensers (i.e., local pharmacists and shopkeepers) were carried out in each village. ICs and POs were documented through detailed fieldnotes. To strengthen the validity of findings, data from the three qualitative components, drug bag interviews, simulated patient visits, and ethnographic fieldwork (POs and ICs) were triangulated.

#### Sampling strategy.

Participants were selected using purposive maximum variation sampling to capture a diverse range of perspectives and experiences related to antibiotic use [23]. The following criteria guided participant selection: (i) Poultry farmers (minimum one respondent per village); (ii) Older adults (aged >60 years); (iii) Adult men and women with at least one child under five years of age; (iv) Men and women living alone for at least three months; (v) Young adults (aged 18–27 years); (vi) Other adults (aged >18 years) not fitting the above categories.

These criteria were informed by findings from earlier phases of the COINCIDE study, which identified demographic differences in antibiotic knowledge related to age, gender, and occupation. Consistent with the participatory approach used in village selection (see ‘Study Site and Setting’), participant recruitment was conducted collaboratively by the research team and key local stakeholders, namely village midwives, health cadres, and sub-village heads in each study location, through joint discussions that drew on local stakeholders’ knowledge of their communities and collective reflection on how potential participants aligned with the study’s sampling criteria.

Participant recruitment strategies for drug bag interviews varied between sub-districts, based on the recommendations of village midwives, health cadres and sub-village heads, who held in-depth knowledge of local cultural norms and community dynamics. For example, in Bendosari sub-district, community members preferred to be contacted in advance, typically via text message or phone call, by village midwives or health cadres, so that interview appointments could be scheduled prior to home visits. In contrast, in Mojogedang sub-district, community members were approached directly in person, based on the advice of village midwives and health cadres, to provide information about the study and invite their participation. Unlike for drug bag interviews, participants in informal conversations and observations were engaged spontaneously in the course of everyday fieldwork, rather than through formal recruitment procedures, reflecting the exploratory and situational nature of ethnographic interactions.

Drug bag interviews were conducted in participants’ homes, allowing researchers to observe living conditions and surroundings (as part of participant observations) and identify and document any leftover antibiotics present in the household. Participant observations of specific aspects of living conditions included general features of the surroundings (e.g., proximity to public roads or clinics), who was present during the interview, whether there were small-scale farms or family businesses on the compound, the general condition of the house, and whether participants lived alone. This was an integral component of the drug bag method and ethnographic fieldwork, which helped enrich the contextual understanding of antibiotic use in household and community settings. The research team (DN and TN in all districts; SYN in Klaten district) were present during all drug bag interviews. In some cases, family members or neighbours were also present, having been invited by the participant. ICs and POs took place in and around participants’ homes, as well as in community settings such as shops, streets, markets, and pharmacies.

Each drug bag interview lasted between 45 and 70 minutes. A total of 36 interviews were conducted across the six villages, guided by the principle of data saturation. Data collection and analysis were conducted iteratively, with data saturation determined through ongoing review of emerging themes in fieldnotes and during weekly debrief meetings, and saturation was reached when additional observations no longer appeared to generate new themes. No participants refused to take part or withdrew from the study. The length of informal conversations varied depending on context and participant availability, ranging from a few minutes during brief encounters to some longer interactions in homes and shops.

#### Research team and reflexivity.

All ethnographic fieldwork was conducted by two Indonesian female researchers (DN, MPH in Health Nutrition with extensive prior experience in qualitative and quantitative health research; TN, BA in Philosophy with professional experience as a journalist and additional training in qualitative methodologies). Both were native to Central Java and fluent in Bahasa Indonesia and Javanese, which supported rapport building and contextual understanding.

The researchers had no prior relationships with study participants. Community access and initial introductions were facilitated by village midwives and health cadres, who were well-known and trusted within the communities. At the beginning of each interaction, the researchers introduced themselves, explained their backgrounds, and described the purpose of the study to establish rapport and trust with participants.

The shared cultural and linguistic background of the local research team facilitated access to informal spaces and everyday interactions in which antibiotic use was discussed and enacted, and made it possible to recognize practices that were locally normative rather than exceptional. At the same time, the researchers reflected on how their educational backgrounds and perceived professional status could shape interactions, such as leading to more socially desirable responses during simulated patient visits and observations in pharmacies and *Warungs*.

Daily debriefings were conducted between DN and TN to reflect on field experiences and emerging themes. Additionally, weekly debrief meetings were held with the qualitative study principal investigator (MLS) and research collaborator, and a PhD candidate (SYN) to support interpretation, reflexive discussion, and preliminary analysis across the broader research team. These reflections informed analytic decisions, particularly in interpreting antibiotic use as a socially embedded and routinized practice rather than as individual misuse, which reflects the key findings of the study. As such, in this study, we were mindful to avoid commonly used terms such as “misuse” and “inappropriate” antibiotic use, which can sound punitive and over-attribute individual agency while underplaying structural constraints; instead, we use terms such as “non-recommended use” and “use inconsistent with clinical guidelines.”

The Consolidated criteria for REporting Qualitative research (COREQ) checklist was used to ensure transparent and accurate reporting of this study (see S2 File).

### Data analysis

#### Qualitative analysis.

Thematic analysis was used to examine qualitative data, including field notes from ICs and POs, as well as summaries of drug bag interviews. Coding was carried out by three members of the research team (MLS, SYN, and WL), on English language field notes and interview summaries. The coding framework consisted of first-level codes assigned to each antibiotic, alongside thematic higher-level codes and a range of sub-codes (see S3 File for the coding tree).

A combined inductive and deductive approach was employed, with some codes being developed inductively from the data, while others were derived deductively based on existing literature and the research team’s prior knowledge of the topic. NVivo software (version 14) was used to facilitate data management and coding.

Although ascertaining participants’ feedback on the coded data was not feasible for this study, preliminary findings were shared and discussed in focus groups during the COINCIDE study closing meeting in May 2025 in Indonesia with a diverse group of stakeholders, including farmers, physicians, veterinarians, laboratory specialists, government officials, policymakers, Puskesmas staff, livestock officers, donor agencies, associations, embassy representatives, and academics. These findings were incorporated into the drafting of policy recommendations for the relevant stakeholders mentioned above which have been shared with national and international organizations including the Ministry of Health, clinicians, poultry associations, WHO, FAO and Fleming Fund.

#### Quantitative analysis.

For each antibiotic category, binary variables were available indicating whether the antibiotic was recognized, ever used, frequently used, used in the past 30 days, or considered difficult to obtain in the past 30 days. These binary responses (coded 1 = yes, 0 = no) were summed within each antibiotic category to obtain absolute counts for each metric. To facilitate comparison between categories, we additionally calculated percentages by dividing the count for each metric by the sum of all five metrics within that category and multiplying by 100. RStudio version 4.1.2 was used for the visualization and statistical analysis of all data.

### Ethical statement

The study received ethical approval from the Faculty of Medicine, Universitas Indonesia - Cipto Mangunkusumo Hospital, Indonesia (number KET-294/UN2.F1/ETIK/PPM.00.02/2022); the National Research and Innovation Agency (BRIN), Indonesia (number B-2109/II/HM.00.01/4/2022); the Institute of Tropical Medicine, Antwerp, Belgium (number 1652/22). The COINCIDE study is registered at www.ClinicalTrials.gov (NCT number NCT05960084).

Prior to fieldwork implementation, the local regional clearance approvals were obtained from the respective districts and sub-districts. Participants of the drug bag interview received detailed information about the study’s purpose, procedures, and their rights, including the right to withdraw at any time without consequence. Verbal informed consent was obtained prior to data collection from all participants taking part in in-depth drug bag interviews. Verbal informed consent was preferred in this study context, because the act of signing one’s name can represent a reason for mistrust, thereby reducing the quality of the data collected. Consent was documented by the lead researcher present at the time by completing the consent form, with the research assistant acting as a witness to the consent. Participants involved in drug bag interviews received a bar of soap and a water bottle as a token of appreciation for their participation.

In line with established ethnographic practice, individual consent was not obtained for informal conversations and observations in public settings, including interactions with pharmacists and shopkeepers for antibiotic purchases during simulated patient visits. These were naturally occurring encounters and routine interactions in public spaces, posing minimal risks to participants, while safeguarding their confidentiality, and could not have been conducted with full disclosure of the researcher’s identity. The study met accepted conditions for ethical permissibility for covert observations, including minimal risk, confidentiality, and the generation of knowledge with significant social and policy value, not obtainable through consented observation [24]. No identifying information about pharmacies or participants involved in simulated patient visits was recorded, and only aggregated data were reported.

The study adhered to the ethical principles outlined in the Declaration of Helsinki. All data were anonymized to ensure participant confidentiality.

## Results

### Pharmacy and *Warung* ethnography

This section presents key findings on the availability of antibiotics without a prescription at local pharmacies and shops (*Warungs*). Table 3 provides an overview of antibiotic purchase attempts during simulated patient visits in each district, showing the number of outlets visited and outcomes of these attempts.

**Table 3:** Summary of antibiotic purchase attempts during simulated patient visits in pharmacies and *Warungs* by district.

District	Outlet type	No. of outlets visited	Sold without prescription	Refused sale	Claimed “out of stock”	Sold with prescription
**Sukoharjo**	Pharmacies	7	5	1	0	1
	*Warung*	6	4	1	1	0
**Karanganyar**	Pharmacies	10	8	1	1	0
	*Warung*	3	2	0	1	0
**Klaten**	Pharmacies	9	5	4	0	0
	*Warung*	7	3	0	4	0
**Totals**	**All types**	**42**	**27**	**7**	**7**	**1**

Overall, success of buying antibiotics without a prescription was the most common outcome among visited outlets, while only a minority of outlets either refused the sale or stated that the requested antibiotic was ‘out of stock’. However, the ease of obtaining antibiotics without a prescription differed across the three study districts. In Sukoharjo district, pharmacy staff frequently asked how long the symptoms had persisted before agreeing to sell antibiotics. In Karanganyar district, antibiotics were most easily accessible without a prescription, with the large majority of outlets asking little to no questions following a purchase request:

*“We asked for ciprofloxacin and she handed out the antibiotics with a very friendly attitude towards us as customers. This was quite surprising as we did not expect less of tense as usual when we visited pharmacy and asked for antibiotics. No questions, no instruction on how to take it, no comment on reluctancy, wrote a receipt of payment. It was very joyful atmosphere, while we obtained the higher class of antibiotics without prescription. This pharmacy, like others, has pharmacies permit printed out on big white board on the wall. We also bought super tetra, only one tablet.”* – Fieldnote IC, simulated patient visit, Pharmacy, Karanganyar district

By contrast, in Klaten district, purchasing antibiotics over the counter was more difficult or sometimes not possible. Among the three districts, Klaten had the highest refusal rate for antibiotic sales, requiring researchers to use more persuasive strategies to bypass prescription requirements (further details are provided below under ‘accessing antibiotics’).

### How do individuals recognize, access, understand and use antibiotics in Central Java, Indonesia?

#### Sociodemographic characteristics of drug bag interview participants.

Sociodemographic characteristics of drug bag participants are summarized in Table 4. The study population was predominantly female (83%) with a minority of male representation (17%). Participants’ ages ranged from 20 to 87 years, with a mean age of 44 years. Most participants were between 20 and 59 years old, with the largest age group being 20–29 years (28%), followed by older participants aged 65 and above (17%), and those aged 30–39 years (22%).

**Table 4:** Participants’ demographic characteristics.

Characteristic	Category	n	%
**Total participants**		36	100
**Gender**	Female	30	83
	Male	6	17
**Age group**	20–29	10	28
	30–39	8	22
	40–49	4	11
	50–59	4	11
	60–64	4	11
	65+	6	17
**Household size**	1	5	14
	2	5	14
	3	3	8
	4 *(mode)*	10	28
	5	9	25
	6	3	8
	7	1	3
**Household role**	Spouse of head of household	16	44
	Head of household	11	31
	Child	7	19
	Relative	2	6
**Education**	None	7	19
	Elementary	6	17
	Middle school	7	19
	High school	9	25
	University graduate	6	17
	University student	1	3
**Main occupation**	Housewife	11	31
	Livestock farmer (all categories)	7	19
	Agricultural farmer	3	8
	No work (retiree)	3	8
	Unemployed	3	8
	Informal worker	2	6
	Others (merchant, nanny, teacher, etc.)	7	19

Education levels were generally low. Among them, 19% had no formal schooling, 17% had completed elementary school, 19% middle school, and 25% high school. Six participants (17%) had completed university degrees in fields such as biology education, nursery, management, informatics management, agriculture, and veterinary science. One participant was a current university student in midwifery.

Regarding household size, households were most commonly composed of four members (range: 1–7 members). A quarter of participants (25%) lived in households of five members, while about 14% lived in smaller households of one or two members. In terms of household roles, spouses of household heads comprised the largest group (44%), followed by heads of household (31%; the person identified by household members or official records as having primary responsibility for managing and representing the household), children (19%), and other relatives (6%). Participants’ occupations and livelihoods reflected rural economic contexts. Housewives represented the largest group (31%), followed by livestock farmers, primarily egg-layer chicken keepers (19%). Other occupations included agricultural farming, petty trade or merchant activities, informal work, factory labour, nanny work, and teaching. Three participants reported being unemployed, while an additional three were retirees with no work.

#### Key findings of the drug bag interviews by antibiotic type.

**Fig 4:**
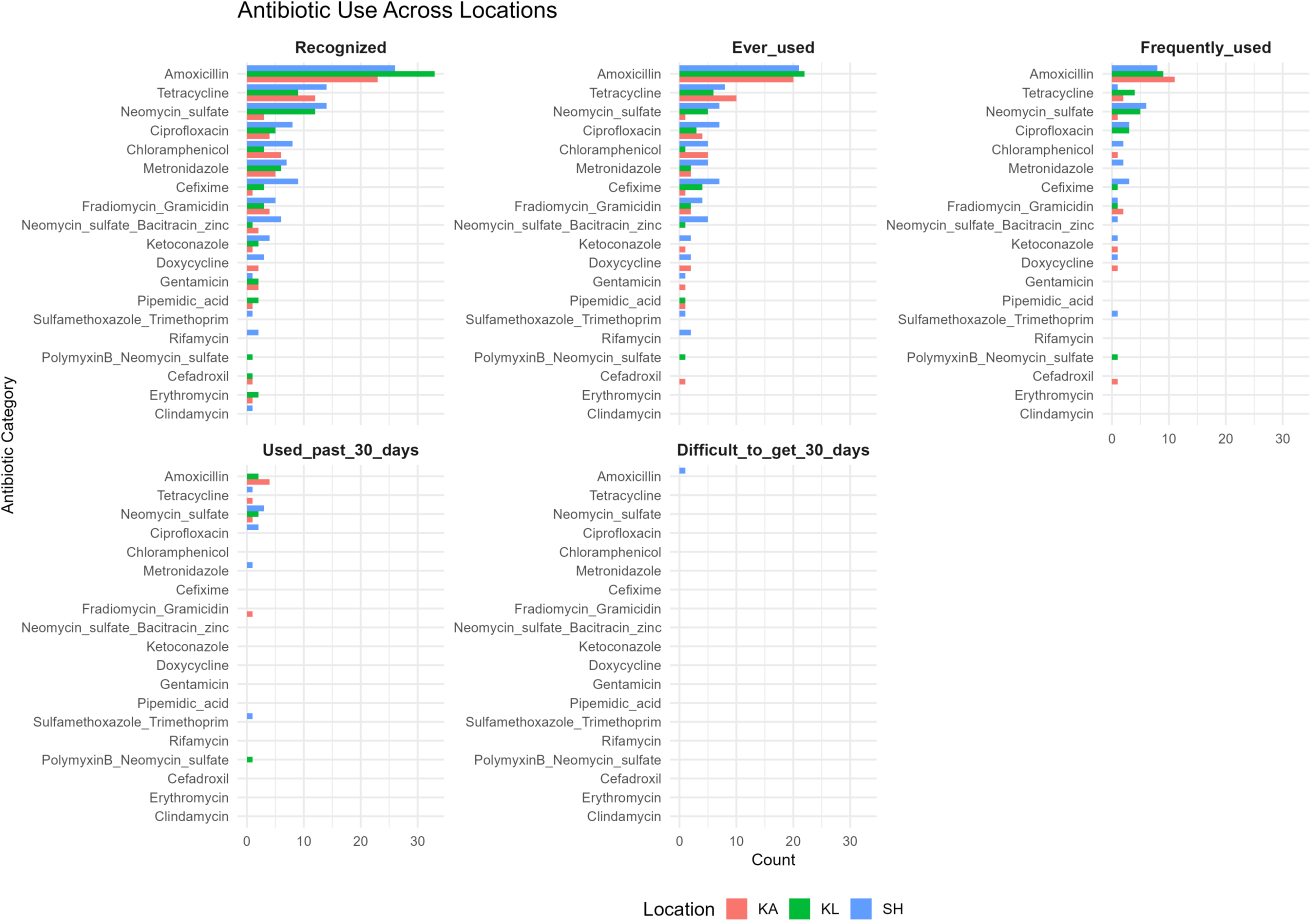
Horizontal bar chart showing how individuals recognize, access, understand, and use antibiotics across three study locations. Each panel represents a different stage of drug bag interview: recognition, ever used, frequently used, used in the past 30 days, and difficulty accessing antibiotics in the past 30 days. Colors indicate different locations: KA (Karanganyar), KL (Klaten), and SH (Sukoharjo).

**Fig 5:**
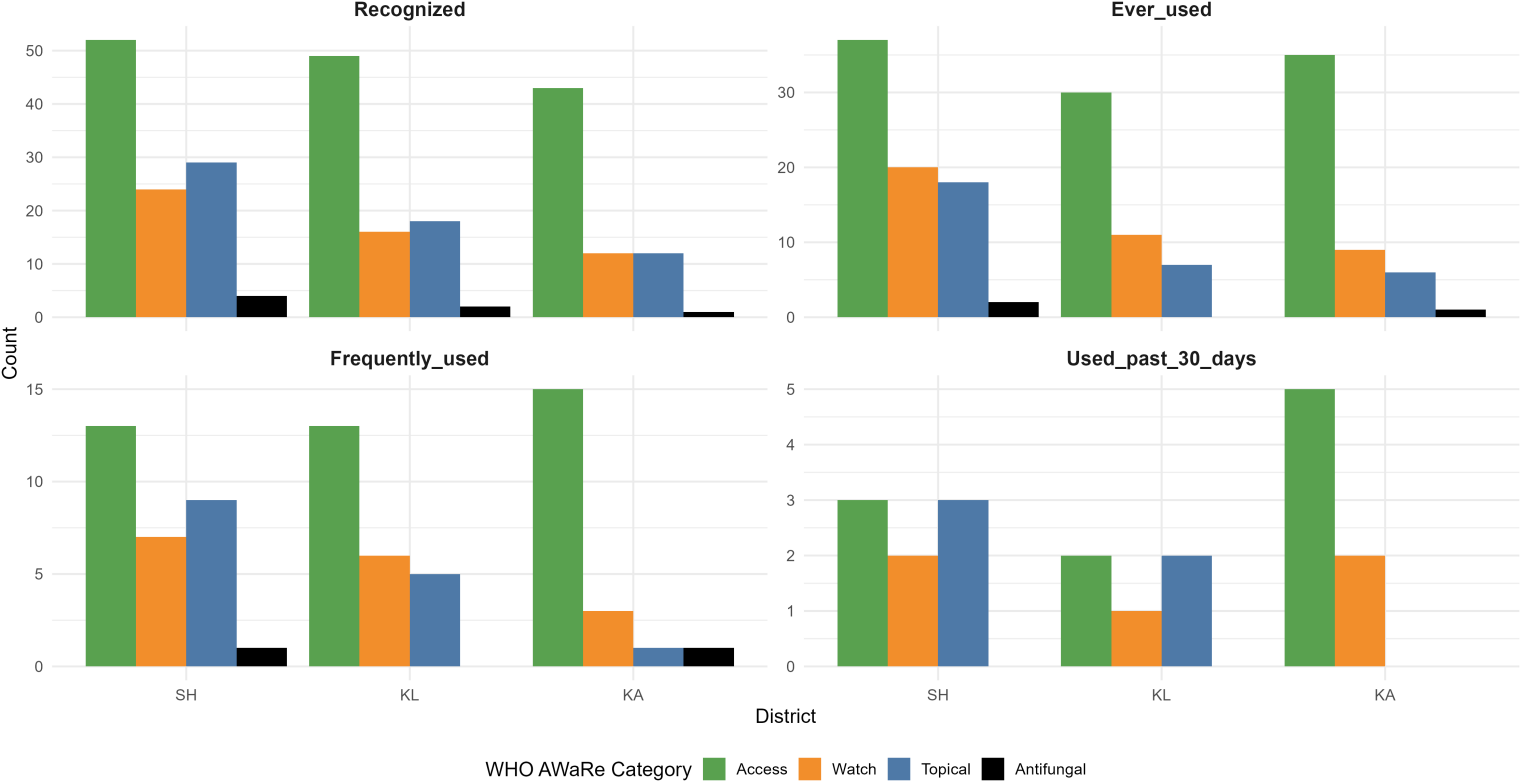
Bar chart based on AwaRe classification. Each panel shows how individuals recognize, access, understand, and use antibiotics based on AWaRe classifications by WHO across three study locations: KA (Karanganyar), KL (Klaten), and SH (Sukoharjo).

**Fig 6:**
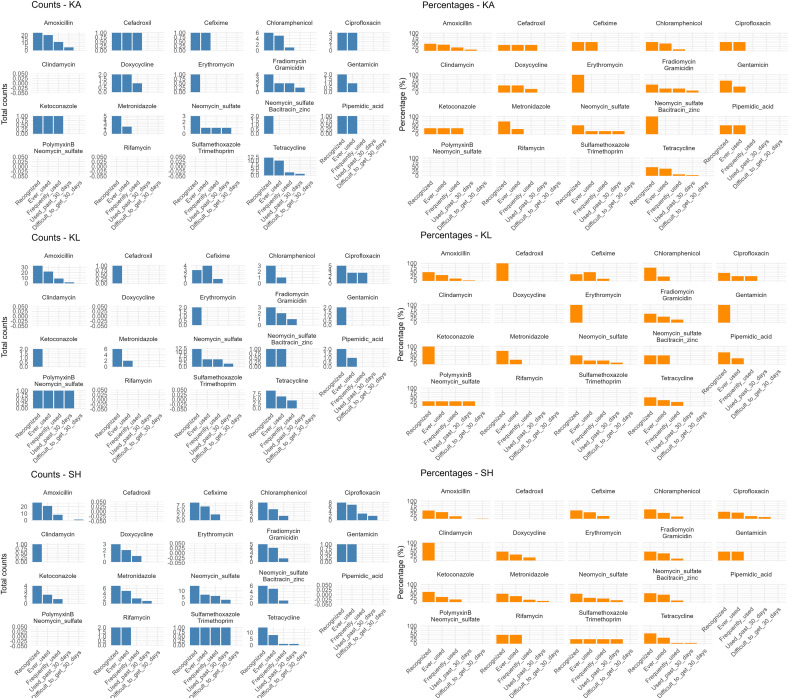
Bar chart of results from five-steps of the drug bag method. Bar chart showing five-step drug bag assessment of antibiotic use in three districts (KA = Karanganyar, KL = Klaten, SH = Sukoharjo). This figure presents the distribution of responses across five sequential steps of the drug bag method: (1) recognition of antibiotics, (2) ever used, (3) frequent use, (4) use in the past 30 days, and (5) difficulty obtaining in the past 30 days. Data are shown for multiple antibiotics across three districts, highlighting patterns of awareness, usage, and access at the community level. Blue bars represent the absolute counts of respondents for each step, while orange bars indicate the corresponding percentages.

Across the three surveyed locations, a total of 45 different antibiotic products were identified, showing diverse patterns of recognition and usage. Amoxicillin-based formulations were the most widely recognized and frequently used, followed by Super Tetra (tetracycline) and topical products such as neomycin sulfate. Other widely known antibiotics included Betason-N (neomycin sulfate), and FG-Troches Meiji also known as Meiji candy containing fradiomycin (neomycin) and gramicidin, though their actual usage varied by location. Across all sites, access to antibiotics was stable, with only minimal reports of difficulty obtaining amoxicillin in Sukoharjo district in the past 30 days (Fig 4). The distribution of antibiotics according to the WHO AWaRe classification showed that the majority of recognized and frequently used antibiotics belonged to the *Access* category, followed by a smaller proportion in the *Watch* group, with no representation from the *Reserve* group. The Sukoharjo district showed the highest recognition and usage of *Watch* category antibiotics. The figure also shows regional differences in topical and antifungal products (Fig 5). The drug bag five-step results from each district are further illustrated in Fig 6.

#### What is an antibiotic.

Participants’ understanding of what constitutes an antibiotic as revealed in drug bag method interviews can be grouped into four broad categories: no knowledge, misconceptions, a mix of correct and incorrect understanding, and accurate knowledge. The majority of participants reported having no knowledge of antibiotics, stating they had never heard of the term or did not know what antibiotics were. Misconceptions were also common and included beliefs that antibiotics are used to prevent illness or infections, including viral infections, or that they function as painkillers, vitamins, or to boost immunity. Several participants exhibited mixed understanding, on the one hand correctly identifying antibiotics as medicines like Super Tetra and amoxicillin used to fight germs and treat diseases from within the body, while also associating them with non-antibiotic medications like paracetamol, and describing their purpose as killing viruses in the body or treating symptoms like fever or cough, or preventing wound infections.

*“In the beginning of the interview, she referred to an antibiotic as paracetamol, to treat fever. Later in the end of the interview, she changed her opinion on what was an antibiotic. She stated that antibiotics was medicine to prevent scab/ulceration”*. – IDI, drug bag participant, female, 51, Sukoharjo district

Only a few participants demonstrated a clear and accurate understanding, correctly identifying antibiotics as medicines used to treat bacterial infections, while being ineffective against viruses.

#### Accessing antibiotics.

Qualitative analysis identified four main patterns related to the strategies used to obtain antibiotics over the counter and the typical outcomes of these interactions. With the exception of one participant (young female college student studying midwifery) who reported purchasing antibiotics online via *Halodoc*, a commonly used telemedicine service in Indonesia, all others obtained them in person within their local community. First, antibiotics were frequently dispensed without a prescription when customers presented at a pharmacy or *Warung*, either by simply requesting a named antibiotic or by describing specific illness symptoms:

*“‘Mbak, minta obat gatal (Miss, I want itchy medicine)’ said one middle-aged man to the shopkeeper, without mentioning the brand or asking how to use it. The shopkeeper also did not educate the buyer on how to use it etc. She just straight away handed out the medication [Gentamicin Sulfate Synalten-(IFARS)] and continued the transaction. Seems like the consumers often buy it here and the shopkeeper already recognized his ‘usual’ medicine.”* – Fieldnote PO, simulated patient visit, Pharmacy, Sukoharjo district

Buying popular antibiotics in smaller quantities such as just one or two tablets was also commonly possible, such as in the case of amoxicillin or Super Tetra:

*“The owner easily handed out amoxicillin, even let us buy only two tablets if we want... This shocked us that we can buy amoxicillin with only two tablets.”* – Fieldnote IC, simulated patient visit, *Warung*, Sukoharjo district

*“We then also asked did she have* Super Tetra *and she said yes. We then asked could we buy one tablet instead of one complete strip (6 tablets). She said yes.”* – Fieldnote IC, simulated patient visit, Pharmacy, Karanganyar district

A second observed trend was an initial reluctance by pharmacy staff to dispense antibiotics over the counter, which was often overcome through persistent requests by the customer or ‘mystery client’. Successful strategies to obtain antibiotics without a prescription included repeating and emphasizing the severity or duration of symptoms, referencing prior experiences where a general practitioner (GP) had prescribed a specific antibiotic for similar symptoms, or naming a known healthcare provider such as a GP, midwife, or hospital staff member:

*“We bought antibiotics at the […] pharmacy under the name of a GP who works at the Puskesmas […] that we met in the morning… The way we are able to obtain the antibiotics is also unique because we only need to say the GP’s name and the staff search the name in the computer and quickly confirm the name and let us buy anything we want.”* – Fieldnote PO, simulated patient visit, Pharmacy, Sukoharjo district

Some pharmacies, rather than dispensing the specific antibiotic requested, preferred selling non-generic branded antibiotics, particularly for generics like amoxicillin known to require a prescription. Although the reasoning was not always made explicit, this strategy appeared to be a workaround to avoid regulatory scrutiny. Furthermore, some pharmacies also requested the customer’s name or phone number before selling antibiotics without a prescription. In such instances, staff were also typically reluctant to issue a receipt, even when explicitly requested by the customer:

*“In the beginning, we asked for cefadroxil, but then she refused to give any antibiotics without prescription. [The researcher] then explained the story of her reoccurring urinary tract infection and how the GP usually prescribed her cefadroxil. [The pharmacist] explained again that it is not allowed to sell antibiotics without prescription. She then offered another type of antibiotic under the name Urinter. She said this medicine is antibiotics too and this is a good alternative… In the end, when we asked for receipt of buying those products, she said that she could not give that because Urinter is considered an antibiotic.”* – Fieldnote PO, simulated patient visit, Pharmacy, Sukoharjo district

Thirdly, some pharmacies refused to sell antibiotics without prescription, offering alternative non-antibiotic medicines instead. However, such refusals were not consistently observed across pharmacies – or even within the same pharmacy – with some antibiotics being sold without prescription, while others were not:

*“We are again become fake patient looking for amoxicillin. She said that she cannot give us antibiotics without prescription. She asked what symptoms or sickness [researcher] had. [The researcher] answered that she has sore throat. She then asked again how long has been already. [The researcher] answered that it has been three days. She then handed out methylprednisolone and said that she only can give me that medicine.”* (Note: this same pharmacy gave out cefixime for another problem). – Fieldnote PO, simulated patient visit, Pharmacy, Sukoharjo district

Finally, at some pharmacies, requests for antibiotics without prescription were met with the response that the antibiotic was “out of stock”, which was particularly often the case when requesting Super Tetra. This may have reflected a genuine stockout, or a polite strategy for refusing an over-the-counter sale. In such cases, customers were often offered alternative medications, sometimes including antibiotics.

#### Awareness and understanding of Indonesian medicine labels.

**Fig 7:**
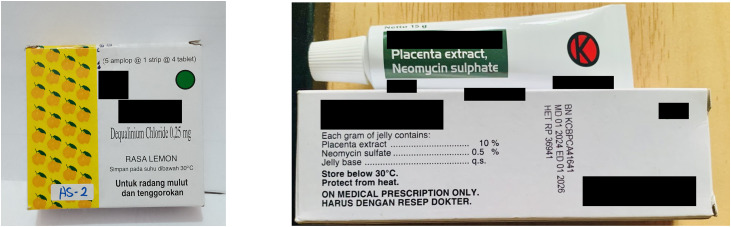
Indonesian medicine packages. Medicine packages showing the official Indonesian medicine labels: (left) the green circle symbolising over-the-counter (OTC) or free drugs (*obat bebas*) and (right) the red circle with “K” symbolising prescription drugs (*obat keras*).

Participants generally demonstrated low awareness and understanding of the coloured dots on medicine packaging, which are part of Indonesia’s regulatory system for classifying medications. Most reported never paying attention to the labels, including the red dot indicating prescription-only medicines such as antibiotics (Fig 7):

Among those who were aware, only a few correctly identified red-labelled medicines as requiring a doctor’s prescription, and green-labelled products as over-the-counter and freely available medications. A few participants interpreted the colours more loosely, associating the colour dots with the strength of the medicine:

*“[She said she] paid attention to red or green circle on medicine packages. What she knew, red indicating it was “obat keras” [“hard medicine”] and green was “obat bebas” [“light medicine”].* – IDI, drug bag participant, female, 24, Klaten district

Despite this partial understanding, all participants reported having purchased red-labelled antibiotics without a prescription, often prioritising reliance on past prescriptions or informal advice from friends and family. While a few participants claimed to avoid such purchases, drug bag interviews revealed contradictions, suggesting limited understanding of which medicines fall under prescription-only categories. Only one participant expressed fears about self-medicating with red-labelled drugs, reflecting some risk awareness. In contrast, while there was some awareness of the halal label on medicine packages, most participants explained that they never paid attention to it, commonly citing trust in doctors as the reason.

#### Reasons for antibiotic self-medication.

Participants identified several reasons for preferring to purchase antibiotics directly from local pharmacies or *Warungs*, rather than visiting a healthcare provider at a local Puskesmas. The most common motivations were convenience, ease of access and lower cost. As one participant explained:

“According to her, her health seeking behavior seemed to rely more on pharmacies... The main reason seemed to be access… and more affordable as they only need to pay for the medicine without paying the consultation session. Puskesmas or midwife practice was quite affordable and free for all BPJS* *card holder, but the opening schedule was inconvenient for her. It was easier to remember what medicine was given by midwife and then buy it by themselves in a nearby pharmacy. Or just visit a pharmacy and say their symptoms and ask for their suggestion of medicines.” –* Fieldnote IC, female, 23, Karanganyar district

*Note:BPJS (Badan Penyelenggara Jaminan Sosial) is Indonesia’s national health insurance program providing universal healthcare coverage.

For some, the perceived benefits of obtaining antibiotics directly via a pharmacy or warung outweighed the perceived value of a medical consultation with a healthcare professional.

*“Cheap medicine that might only cost IDR 1,000 might give better result compared to spending more money for doctor consultation”.* ***–*** Fieldnote IC, female, 48, Karanganyar district

A small number of participants additionally mentioned not being satisfied with the outcome of Puskesmas visits, as they felt they had less choice over the treatment received than when visiting a pharmacy or *Warung*:

*“So far, she had visited Puskesmas [name] three times and every single time, Puskesmas [] gave the same skin ointment [containing tetracycline]... She was not satisfied with the treatment by Puskesmas. She wanted to change the medicine or ask doctor to re-evaluate her condition, but she was afraid of being scolded. She saw that Puskesmas usually insisted on asking patients to try the medicine first and see how it goes”.* – IDI, drug bag participant, female, 29, Sukoharjo district

Only very few participants expressed concern over antibiotic self-medication and a preference for prescription antibiotics, perceiving over the counter antibiotics to be unsafe. In particular, participants reported their concerns regarding mixed medicine bags:

*“[He] knew there were places that sold those mixed medicines. But he never saw it or bought it… Because he thought that with these kinds of medicines it would be hard to know what was inside it. It was better to go to GP so they could get further consultation in case something goes wrong with it.”* – IDI, drug bag participant, male, 60, Karanganyar district

#### Stopping antibiotic treatment early.

Participants commonly reported stopping antibiotic treatment once symptoms had improved. Even when the medication had been prescribed with instructions to complete the full course, participants often explained that there was no need to continue the medication once they felt better. Many participants explained that rationing antibiotics in this way enabled them to ‘save’ leftover antibiotics in the home for future use, either for themselves or family members:

*“[The participant] said that on average she and her family did not finish the medicine they had to take, if they felt cured they would stop taking the medicine.”* – IDI, drug bag participant, female, 38, Karanganyar district

Only one participant expressed concerns about taking antibiotics “for too long,” citing fears of potential harm. Two participants also recalled being told by doctor and a nurse, respectively, that it was unnecessary to finish a prescribed course of antibiotics, with this incorrect guidance influencing their use behaviour:

*“[She] remembered that she only took Amoxicillin for one day and then stopped. Doctor never said that this medicines should be finished, instead, according to [her] the doctor said that she could stop taking medicine once the diarrhea had stopped.”* – IDI, drug bag participant, female, 26, Karanganyar district

#### Repurposing antibiotics.

Several participants described the practice of repurposing oral antibiotics for topical use by opening capsules and applying the liquid or powder contents directly onto infected skin wounds. This practice was reported specifically for antibiotics such as amoxicillin and Super Tetra, and was described as traditional knowledge, which is locally developed and culturally embedded, rather than learned from formal education, passed down through family members or neighbours, sometimes across multiple generations:

*“She used [*Super Tetra *(tetracycline)] for skin irritation such as itchiness or external wound due to knife cut etc. She squeezed the paste inside* Super Tetra *capsule using needle or pin to open the capsule. She used it around three times directly onto skin or wound.”* – IDI, drug bag participant, female, 40, Klaten district

A few participants also described using this practice among her animals:

*“She and her husband used* Super Tetra *to treat their cows whenever it had open wounded. They used* Super Tetra *as a skin ointment for cows’ open wound. The frequency of putting the paste of* Super Tetra *was not consistent, she only said they gave it to the cows until the wound healed.”* – IDI, drug bag participant, female, 65, Karanganyar district

#### Leftover antibiotics and sharing with family members.

Many participants reported storing leftover antibiotics at home for future use, using them interchangeably among family members experiencing similar symptoms, without first consulting a healthcare provider. This behaviour was said to be driven by perceived symptom similarity, convenience, and past experiences with the same illness. One participant, for example, described reusing antibiotics obtained for their mother’s diarrhoea to treat a sibling with similar symptoms later on, combining leftover and newly purchased supplies:

*“The medicine that was used last month by her mother and brother was cotrimoxazole. They shared the medicine. They obtained it from the GP for her mother’s diarrhea and when her brother experienced diarrhea as well, they bought it again by themselves from nearby pharmacy… Her brother finished the whole strip plus the leftover from her mother.”* – IDI, drug bag participant, female, 20, Sukoharjo district

In some instances, participants reported throwing away unused antibiotics and one outlier participant explained that they explicitly avoided sharing antibiotics due to concerns about incorrect dosing, stating that prescription antibiotics were safer. However, examples of such cautious behaviour were rare, while overall awareness of the risks associated with antibiotic sharing appeared to be limited.

*“[The participant] kept quite a lot of supply of medicine at home which she stored in plastic containers. Most of these drugs are leftover drugs that she saves to take again if needed.”* – IDI, drug bag participant, female, 38, Karanganyar district

#### In the spotlight: four antibiotic case studies.

Across all study sites, four products stood out for their consistent and widespread use: amoxicillin, Super Tetra, topical aminoglycoside creams, and oral FG Troches Meiji. These medicines were not only frequently recognized and accessed, but also commonly used in everyday treatment practices. In addition, we found the presence of pipemidic acid (Urinter), a drug considered high-risk and restricted in European settings. These four distinct patterns became central to our ethnographic investigation and are explored in the following case studies (see Boxes 1–4).

**Box 1. Amoxicillin: the Coca-Cola of antibiotics.**

Amoxicillin is like the Coca-Cola of antibiotics; widely known and easily found in our study area, often without a prescription. People often call it “amoxcilin” and it is sold as pills or syrup. People can buy it in small quantities, just one or two tablets if that’s all they need. In some “by the book” pharmacies refuse to sell generic amoxicillin without a prescription, they usually offer branded versions instead (e.g., Yusimox). During our fieldwork, we came across many cases where people kept leftover amoxicillin at home, the same casual way people keep painkillers like paracetamol or ibuprofen at hand. Sharing amoxicillin with family members was also very common. Hardly anyone seemed to notice the big red “K” printed on amoxicillin packages, Indonesia’s official symbol for prescription-only drugs. This example of amoxicillin shows that there is still a long way to go before antibiotics are used more prudently in everyday life. (Fieldnote and reflection MLS July 2024).

**Box 2. Hereditary wound medicine: Super Tetra.**

I spoke with an older woman who described Super Tetra as a “hereditary medicine” in her family. She said her parents and grandparents used it both on wounds and orally, passing down the practice through generations. According to her, the paste inside the capsule is squeezed out and applied directly on wounds because they believe it helps wounds heal faster. This method is used not only for people but also on chickens and other animals.

Super Tetra remains very popular and easily accessible. People often buy just one or two pills at a time, sometimes from informal sellers. However, it has become harder to find recently. A local reseller explained that they get supplies through a personal contact and that the price has risen from IDR 17,000 to IDR 18,000 per tablet (approximately € 0.89-0.94).

Dosage is not consistent; people use Super Tetra as they feel necessary. Its use is embedded in the community’s everyday healing practices and remains an important, trusted medicine for external wounds and other ailments. (Fieldnote DN & TN Dian PO IC October 2024).

**Box 3. Urinter (pipemidic acid) use should be banned in Indonesia.**

Urinter, which contains pipemidic acid, is widely used in Indonesia to treat urinary tract infections (UTIs) and can be easily purchased without a prescription. However, there is growing recognition that pipemidic acid should be banned for human use in Indonesia as it has been in many other countries, including in Europe. Pipemidic acid belongs to the class of quinolone antibiotics and shares similarities with other drugs that have been linked to serious side effects such as tendon damage, nerve disorders, and increased risk of antimicrobial resistance. Because of these risks, regulatory authorities in Europe have restricted or banned its use.

In Indonesia, the continued availability and widespread over-the-counter sale of Urinter may contribute to use inconsistent with clinical guidelines, including self-medication without proper diagnosis, irregular dosing, and incomplete treatment courses. Such non-recommended use not only risks individual patient safety but also accelerates the development of antimicrobial resistance. Field observations showed that many people rely on Urinter to quickly relieve symptoms without accessing formal healthcare. This practice combined with lack of prescription oversight, makes the use of pipemidic acid particularly risky. Stronger regulation and public education about the risks of antibiotics like Urinter (pipemidic acid) are urgently needed to protect communities from harm associated with non-recommended use of antibiotics. (SYN Fieldnote and reflection Oct 2024).

**Box 4. Street-Level remedies for itchiness, wounds, and sore throat: Neomycin Sulfate (topical), Bioplacenton, and FG Troches Meiji (oral).**

In Central Java, FG Troches Meiji, commonly known as “Meiji candy for the throat”, or locally as “*permen bolong*” (hole candy) are widely recognized and often found in almost every household. These small, sweet pills are popular throat lozenges because they taste and look like candy but actually contain two important antibiotics: fradiomycin (a form of neomycin) and gramicidin. They are used widely for sore throats, cough, cold, throat inflammation, and in some cases, regarded as a vitamin or even “ulcer candy”. Many bought FG Troches Meiji without prescriptions from pharmacies and occasionally from *Warungs*. The thin boundary between medicine and confectionery is not clear. During fieldwork, I surprisingly found one member of our research team casually opening a pack and consuming FG Troches Meiji like sweets. This situation shows just how casually Meiji candy has become part of everyday life. (Fieldnote and reflection SYN Oct 2024).

In this community, medicines like neomycin sulfate ointment and Bioplacenton gel are part of everyday life. They are the go-to for common skin problems such as redness, itching, wounds, and burns. For example, one woman told us how she used Bioplacenton for a wound after falling. She bought it at the clinic’s pharmacy but never saw a doctor first. She only applied it once a day while cleaning the wound and didn’t always finish the ointment, sometimes throwing the rest away. Others said they used Bioplacenton many years ago for burns or skin wounds because it “restores the skin” and helps it heal fast.

Neomycin ointment is well known for stopping itchiness and helping dry or wet wounds. Many caregivers apply it to their children’s skin multiple times a day, often after bathing or before bed. When the skin is very sore, they sometimes sprinkle baby powder first to prevent burning from the ointment. Even though the packaging says to avoid long use and doctor prescriptions, most people buy these ointments directly from pharmacies, trusting their own knowledge or neighbors’ advice. (Fieldnote and reflection DN & TN Oct 2024).

## Discussion

This ethnographic study used participant observations, informal conversations, simulated patient visits, and drug bag interviews to explore how community members recognise, access, understand and use antibiotics in everyday life, as well as the wider social and cultural factors influencing antibiotic use in Central Java, Indonesia. We found that antibiotics were widely accessible without a prescription through both pharmacies and *Warungs*, and that their use is deeply embedded in daily health practices. These findings highlight how antibiotic use behaviours are shaped not only by regulatory gaps but also by social norms, economic considerations, and local knowledge. For example, participants commonly described antibiotics as a cheaper and more convenient option than visiting clinics or specialists, particularly when factoring in consultation fees. In addition, decisions about antibiotic selection and dosing were often guided by advice from family members, neighbours, or shopkeepers, and by prior personal experience, rather than by biomedical knowledge.

### Over the counter availability and access

Findings from this study showed that antibiotics were still widely available and informally accessible in Central Java province despite existing regulations. Almost one third of our simulated patient visits resulted in the successful purchase of antibiotics without a prescription, confirming that antibiotics were obtainable through both formal outlets (pharmacies) and informal settings (*Warungs*). These findings are consistent with national monitoring data from the Indonesian National Food and Drugs Authority (BPOM), which has reported alarmingly high rates of dispensing antibiotics without prescription (70% dispensing rate), and results from other LMICs, where regulatory enforcement is often weak and antibiotics are treated as everyday household commodities [3,25]. In line with our findings, evidence of high rates of antibiotic purchase with non-prescription (87–100%) and antibiotic self-medication (20–100%) was also shown in a systematic review on AMU studies conducted in Indonesia [17]. Together, these findings highlight weak regulatory enforcement and the need for stronger surveillance and accountability at the retail level.

### Poor knowledge, misconceptions and self-medication

Our drug bag ethnography further highlights the ways in which antibiotic use is deeply embedded within social and cultural dimensions of people’s everyday lives. Rather than being guided by public health frameworks, everyday antibiotic practices are largely shaped by past experience, familial traditions and local norms. Community members showed varying levels of understanding of antibiotics, with the majority showing complete lack of understanding and only very few demonstrating accurate knowledge. Similarly, pooled data presented by Limato et al. (2022) showed a substantial lack of AMR awareness and knowledge; with 23–26% of participants not knowing that antibiotics treated bacterial infections and 58–74% stating that antibiotics can cure viral infections; and antibiotic knowledge being associated with higher education and higher income [17]. This variability reflects the absence of consistent public health messaging, particularly on the concept of antibiotics and resistance, the language used to describe them, and the dominance of informal channels of information, including family, neighbours, and local vendors. These knowledge gaps contribute to common misconceptions, such as using antibiotics for viral infections, as painkillers, or immune boosters and shape practices such as poor-adherence to treatment courses, sharing antibiotics within a household, and storing leftovers for later use.

Our findings further showed that health-seeking behaviours for over the counter antibiotics were primarily driven by factors of convenience, easy access from pharmacies and *Warungs*, as well as lower cost and higher patient satisfaction compared to seeking care from formal healthcare settings such as Puskesmas and hospitals. These findings are aligned with studies conducted in similar settings, where self-medication is often driven by positive prior experiences and practical considerations, including ease of access and cost [17]. This reaffirms that antibiotic use is not only a matter of knowledge but also of structural barriers and lack of trust in the formal healthcare system.

### Topical and everyday antibiotic use

Amoxicillin, tetracycline (widely known as Super Tetra), and aminoglycosides (used both topically and as troches) were the most frequently used antibiotics in our study localities. Our findings further showed that dermatological conditions such as itching and eczema appeared to drive this demand. Remarkably, polymyxin B, similar to colistin (polymyxin E) topical cream was also found during the drug bag interviews in Klaten district, while *Reserve* antibiotics such as polymyxin should not be included in topical formulations. A similar study conducted in Ghana reported extensive use and potential overuse of topical antibiotics, and likewise emphasized the need to include topical antibiotic use in comprehensive antibiotic stewardship programs [26]. The use of antibiotics in topical preparations, such as aminoglycoside and colistin, both important antibiotics to treat infections by multidrug-resistant pathogens, is not currently classified under the WHO AWaRE framework. The widespread use of topical and combination products containing antibiotics and steroids in Indonesia highlights an urgent gap in global monitoring and stewardship strategies, which should be addressed as part of the national strategies to slow the spread of AMR.

### Repurposing antibiotics and other hidden risks of common antibiotic practices

A striking finding in our study is the practice of repurposing oral antibiotics for topical application. As described by participants, this involves opening oral capsules of drugs like amoxicillin and tetracycline capsules (Super Tetra) and applying their contents as a powder or paste directly onto skin wounds. This practice, often described as traditional knowledge passed down through generations, also extends to animals (e.g., treating open wounds on chickens). This finding is consistent with results from a study conducted in Surabaya, Indonesia, which reported that the majority of pharmacy staff (92%) described Super Tetra capsules as a remedy for wound relief, and more than half recommended applying the medicine directly into wounds [27]. While such practices were regarded as practical and unproblematic by participants, repurposing antibiotics in this way represents a significant point of concern for AMR, as it involves unknown dosages and pharmacokinetics, potentially creating a selective pressure for resistant bacterial strains. These findings highlight that, while regulations are often focused on more renowned drugs like amoxicillin, less-known, topical, or unconventional formulations of antibiotics are being overlooked, despite their likely significant contributions to the AMR problem.

Our case studies of specific antibiotics further illustrate the complexity of AMU and AMR in Indonesia. The use of pipemidic acid (Urinter) for urinary tract infections is particularly risky given its disabling and potential permanent side effects, which led to its restriction for human use in Europe in 2018 [28]. Its continued availability in Indonesia, combined with a lack of prescription oversight, necessitates urgent public education and regulatory action. In the case of urinary tract infections, cheap and effective antibiotics such as nitrofurantoin and fosfomycin are suitable alternatives for replacing pipemidic acid. Thus, the Indonesian Food and Drug authority should reevaluate the market registration of risky antibiotics such as pipemidic acid, aligning with the European ban on their use and ensuring equitable access to effective alternative antibiotics.

Furthermore, “candy-like” medicines such as FG Troches Meiji, containing aminoglycosides, pose a threat by their deceptive appearance and taste, which encourage their casual use without medical guidance, as evidenced in our study. Similarly, studies in other countries have documented marketing and advertising techniques used to trivialise and normalise antibiotic use, including using pharmaceutical promotions [29], financial bonuses and free samples [30], using and perceiving antibiotics like sweets or candy [31], or using packaging aimed at children (e.g., cartoons, like the “jeep car medicine” in Myanmar) [8]. Such tactics are likely to undermine public health messages to promote prudent antibiotic use, highlighting the need for stricter regulation of antibiotic branding and advertising. In addition, our findings suggest that without further public education campaigns, the Indonesian medicine labelling system, including the red “K” symbol for prescription-only antibiotics, has limited impact, as most participants were either unaware of or simply disregarded these warnings.

### Policy implications

Together, these findings point to the need for stricter enforcement of regulations and targeted public health campaigns on medicine labels, including antibiotic labelling and prudent use, including education initiatives delivered through social media. However, in order to be effective, such campaigns need to be accompanied by broader policy changes that address the structural and contextual factors underpinning non-recommended antibiotic use and overconsumption of antibiotics. Social, economic, and health-system constraints, such as high disease burden, poverty, and weak health infrastructure, strongly influence both antibiotic prescribing and consumption practices [32], which help explain why behaviour change interventions alone have limited effectiveness. A recent systematic review of qualitative studies from South Asia underscores this point, showing how antimicrobial use inconsistent with clinical guidelines is sustained by interconnected influences across individual, provider, clinical, community, and policy levels within the wider socio-ecological system in which health decisions are made [29]. Addressing non-recommended antibiotic use therefore requires moving beyond a focus on individual behaviour to interventions that also engage with the broader socioeconomic, institutional and policy contexts in which such practices take place.

## Conclusion

Our findings from Central Java show that antibiotics are not only widely accessible without a prescription but are also deeply embedded in everyday life, from practices of self-medication, ‘hereditary’ repurposing and sharing to the casual use of topical and candy-like antibiotics. These practices illustrate how antibiotics are treated as household remedies rather than regulated medicines, pointing to the need for antimicrobial stewardship programs in Indonesia that extend beyond hospitals and formal healthcare facilities to engage with pharmacies, *Warungs*, and everyday community practices, while also strengthening regulation and public education. By highlighting how cultural norms and local practices shape antibiotic use in LMICs, this study contributes to the global efforts to reduce AMU and underscores the importance of tailoring antibiotic stewardship programmes to community realities.

## Supporting information

S1 File Topic guide. (DOCX)

S2 File COREQ Checklist. (DOCX)

S3 File Coding tree. (PDF)

S1 Checklist Inclusivity in global research. (DOCX)
