# Correction: “Miss, I want itchy medicine”: Understanding what, why, and how antibiotics are used in Central Java province, Indonesia through the drug bag method

**DOI:** 10.1371/journal.pgph.0006656

**Published:** 2026-06-09

**Authors:** The PLOS Global Public Health Staff

The first two authors, Soe Yu Naing and Mira L. Schneiders, should be noted as contributing equally to this work.

The publisher apologizes for the error.
