# Supplementary material for: “Miss, I want itchy medicine”: Understanding what, why, and how antibiotics are used in Central Java province, Indonesia through the drug bag method
Source: PLOS Glob Public Health. 2026 Mar 5;6(3):e0005933. doi: 10.1371/journal.pgph.0005933 (PMC12962538; doi:10.1371/journal.pgph.0005933)
Supplement: S1 File — (DOCX) [file pgph.0005933.s001.docx]

***Topic Guide- IDI Drug Bag Method COINCIDE***

1. Date: ______
2. Interviewer name ________
3. Participant: ID ______________
4. Regency ___________
5. Village name ____________

**Take informed consent. Start the recording**.

Respondent characteristics:

1. Age: _____ years
2. Sex
   1. Male
   2. Female
3. Primary occupation of respondent
4. Farmers in aquaculture sector
5. Livestock farmers
6. Agricultural farmers
7. Healthcare professionals
8. Professionals working with small children (example: nanny, teachers)
9. No work
10. Informal worker, labourer (e.g. not in farming, healthcare or with children)
11. Office worker
12. Merchant (e.g., in traditional market)
13. Other
14. Highest level of education of respondent
    1. None
    2. Preschool
    3. Kindergarten
    4. Elementary
    5. Middle school
    6. High school
    7. University & above
    8. Vocational
    9. Don't know
15. Number of people living in respondent’s household: _____ (number)
16. Role of respondent in the household
    1. Head of household
    2. Spouse of head of household
    3. Primary caregiver
    4. Child (>18 years)
    5. Relative
    6. Friend
    7. Other (please specify) ________

**INSTRUCTIONS**: Empty the drug bag and place all medicines on table/on floor in front of participant. Then introduce yourself and explain to participants:

“We would like to show you some medicines that we have brought with us. We would like to ask you some questions about these medicines. Doctors often call these kinds of medicine ‘antibiotics’.

We would like to do an exercises with you, where we will ask you to sort these medicines into different ‘piles’. The exercises are not to test your knowledge, but to find out whether you have seen any of these medicines before and which you have used. There are no right or wrong answers, we are just interested to learn about your experience of using some of these medicines. The reason that we brought these medicines today is to actually show you these medicines, which might help you remember which ones you have seen and tell us more about them.

If you are OK to continue, we will begin.”

**All medicines on table:**

1. **Sorting medicines activity one:** Which of these medicines have you seen or heard of before? Please pick out the ones that you have seen or heard of before and put them into one pile.

Hint: *Encourage participant to pick up each medicine packet one by one, to look at and classify (they can also open it)* (Take a photo)

🡪 among pile ‘recognise’, ask:

1. **Sorting medicines activity two:** Which of these medicines have you or one of your household members ever used before in your household? Please place them together in a pile. (Take a photo)

🡪 among pile ‘ever used’, ask:

1. **Sorting medicines activity three:** We would now like you to pick out the medicines that you use frequently when someone in your household is sick. Please place them together in a pile. (Take a photo)

🡪 among pile ‘ever used’ (i.e. combine ‘frequently used’ and the leftover pile - ‘rarely used pile’), ask:

1. **Sorting medicines activity four:** Which of these medicines did you or one of your household members use in the last 30 days (i.e. 1 month)? (Take a photo)

🡪 among pile ‘ever used’ (i.e. combine ‘used in last 30 days’ and the leftover pile – i.e. ‘not used in last 30 days’), ask:

1. **Sorting medicines activity five:** For each of these medicines, has there ever been a time when you have needed this medicine but could not get it? Please pick out any medicines that you have needed before in your household but you could not get. Please place them together in a pile and tell us a bit about each one you could not get. (Take a photo)

Hint*: Encourage the participant to narrate to you the stories behind this pile (e.g. the story of not being able to access a particular medicine)*

🡪 among pile ‘ever used’:

Specific questions to ask for each medicine from the ‘ever used’ pile (from sorting medicines activity two). Ask all the below questions (12-22) **for each medicine** from this pile, one after the other:

1. What do you call this medicine?
2. Thank you. Can you tell me a little bit more about this medicine please? (allow participant just to talk openly and freely first. If they already answer some of the questions below, that’s great and you can skip those questions in the follow up. Otherwise continue with all questions below)
3. What kind of health problem do you use this medicine for?
4. Probe: *preventive use* – e.g. types of illnesses/symptoms which they are trying to prevent; versus *curative use* – e.g. type of illness/symptoms experienced which they are trying to treat
5. In your opinion, what is the best way to take this medicine? (probe: dosage, schedule)
6. Do you sometimes share this medicine among your household members members, friends or neighbours?
7. Probe (if yes): Tell me a bit more about that. What are the reasons for sharing this medicine with others?

Thank you. I now would like to learn more about how you usually access this medicine:

1. How easy or difficult is it for you to buy this medicine?
2. Where do you usually get this medicine from?
3. Are there other places that you know where you can get this medicine?
4. Probe (if these are not mentioned already by participant): what about from the Puskesmas, private health facility, pharmacy, local shop, online shop, traditional healer, any others?

**Repeat questions 12-22 for each package of medicines in the ‘ever use’ pile**. Once you have asked these questions for each medicines in the ‘ever use’ pile move on to questions 23-30.

**Other general questions:**

Thank you, that’s really interesting and helpful. I have just a few more general questions to ask before we finish the interview.

1. Do you sometimes use medicines that are mostly used to treat animals yourself?
2. Probe (if yes): tell me a bit more: which medicine(s) is that? And what is the reason you use it yourself?
3. Do you sometimes give medicines that are mostly used for humans to your animals?
4. Probe (if yes): tell me a bit more: which medicine(s) is that? And what is the reason you use it for your animals?
5. Thank you. The medicines we brought today belong to a special category of medicines called ‘antibiotics’. Have you heard the term ‘antibiotics’ before?
6. Probe, if yes: In your opinion, can you describe what an antibiotic is?
7. Antibiotic medications sometimes has little symbols on the box (red colour “K” and green colour circle) (show an example at this moment to the participant). Are you familiar with this symbol?
   1. Probe, if yes: Do you pay attention to this symbol when buying medicine?
8. Is there anything else you would like to tell me about using any of the medicines we have brought today?
9. Is there something else in general you would like to share?
