# Supplementary material for: “Miss, I want itchy medicine”: Understanding what, why, and how antibiotics are used in Central Java province, Indonesia through the drug bag method
Source: PLOS Glob Public Health. 2026 Mar 5;6(3):e0005933. doi: 10.1371/journal.pgph.0005933 (PMC12962538; doi:10.1371/journal.pgph.0005933)
Supplement: S3 File — (PDF) [file pgph.0005933.s003.pdf]

Untitled

Codes

Name	Description	Files	References
access		5	21
need prescription		3	13
out of stock		3	14
without prescription		3	36
amoxicillin		34	120
access		26	50
dosage		20	33
side effect		2	2
symptom		15	30
time		8	11
amoxicillin syrup A1.3		17	33
animal antibiotics		9	18

Name	Description	Files	References
Betason-Neomycin, G1.3		8	12
Bioplacenton G1.7		5	9
C1.1		1	3
Cefadroxil D2		5	5
Cefadroxil dry syrup D2.1		4	5
Cefixime (G) - Dexa C1		4	5
Cefixime C2		6	10
Ciprofloxacin (G)-Hexpharm D1		5	10
CM-1		2	2
D1.1		1	1
Enbatic(powder) G1.4		8	18
Erladerm-n G1.1		2	3
F1.1		1	1
F1.2		1	1
family sharing situation		8	12

Name	Description	Files	References
G1.5		1	1
G1.6		1	1
H1.1 FG throces(Meiji)		14	35
dosage		7	7
symptoms		8	9
time		1	1
K1.1		10	17
K1.2		2	3
Kalcinol (ointment)		1	1
ketoconazole		1	2
LASA(look alike sound alike)		12	19
local term		6	7
amoxicillin		2	2
Meiji		2	2
M1.1		1	1

Name	Description	Files	References
Metronidazole E1		7	12
N1.1		1	1
P1.1 Urinter		5	8
Pi Kang Shuang G1.2		10	25
Primavon (Zenith Pharmaceuticals) Q1.1		1	6
Reco ear drop K1.3		4	8
Sriti AS-1 (topical antiseptic cream)		8	16
Supertetra		34	100
access		0	0
symptoms		12	14
usage		7	9
TA-1 (non-steroidal anti-inflammatory drug)		1	3
TA-2		2	2
what is an antibiotic		28	36

Codes\\cross-consumption between human and animals

Name	Description	Files	References
animal medicine to human		2	2
human medicine to bird		1	1
human medicine to chicken		7	10
human medicine to cows		1	2
no crossconsumption between animals and humans		20	20
no crossconsumption between chicken and human		4	4

Codes\\Mira's additional coding

Name	Description	Files	References
Pharmacies first reluctant to hand out AB	(but do so after persistence)	1	4
Pharmacies refuse AB wo prescription	Usually offering alternatives instead	1	5
Price and financial considerations		1	1

Name	Description	Files	References
Stock variability at pharmacies & shops		1	2
Success in buying AB wo prescription	tactics incl.: Customers seek medicine by describing their symptoms/ referring to GP name/ just asking	2	5

Codes\\Mira's additional coding\\Quant coding

Name	Description	Files	References
1 Ever seen-recognise		7	13
2 Ever used		7	11
3 Frequently used		6	6
4 Used in last 30 days		7	7
5 Could not access easily		7	7

Codes\\Mira's additional coding\\Thematic codes

Name	Description	Files	References
AB better than medical consultation		1	1
Good quotes		2	2

Name	Description	Files	References
Knowledge of Indo med labels		29	32
Mixed medicine bag		2	2
prescription AB are safer		2	2
puskemas doesnt give what you want		7	8
Repurposing AB		11	14
Stopping treatment early		7	14
STRONG meds		4	4

Codes\\SoeYu's additional coding

Name	Description	Files	References
Bahasa_names_for_medicine		5	11
Good practices		3	8
Leftover medicines		7	7
No receipt Ab sales		2	5
Symptoms-based association		4	4

Name	Description	Files	References
itching		6	8
Telemedicine		1	2
